# Voltage-Gated K^+^/Na^+^ Channels and Scorpion Venom Toxins in Cancer

**DOI:** 10.3389/fphar.2020.00913

**Published:** 2020-06-18

**Authors:** Alexis Díaz-García, Diego Varela

**Affiliations:** ^1^LifEscozul Chile SpA, Santiago, Chile; ^2^Millennium Nucleus of Ion Channel-Associated Diseases (MiNICAD), Universidad de Chile, Santiago, Chile; ^3^Program of Physiology and Biophysics, Faculty of Medicine, Institute of Biomedical Sciences (ICBM), Universidad de Chile, Santiago, Chile

**Keywords:** cancer, ion channels, scorpion venom, toxins, voltage-dependent

## Abstract

Ion channels have recently been recognized as novel therapeutic targets in cancer research since they are overexpressed in different histological tissues, and their activity is linked to proliferation, tumor progression, angiogenesis, metastasis, and apoptosis. Voltage gated-potassium channels (VGKC) are involved in cell proliferation, cancer progression, cell cycle transition, and apoptosis. Moreover, voltage-dependent sodium channels (VGSC) contribute to decreases in extracellular pH, which, in turn, promotes cancer cell migration and invasion. Furthermore, VGSC and VGKC modulate voltage-sensitive Ca^2+^ channel activity by controlling the membrane potential and regulating Ca^2+^ influx, which functions as a second messenger in processes related to proliferation, invasion, migration, and metastasis. The subgroup of these types of channels that have shown a high oncogenic potential have become known as “oncochannels”, and the evidence has highlighted them as key potential therapeutic targets. Scorpion venoms contain a high proportion of peptide toxins that act by modulating voltage-gated Na^+^/K^+^ channel activity. Increasing scientific data have pointed out that scorpion venoms and their toxins can affect the activity of oncochannels, thus showing their potential for anticancer therapy. In this review, we provide an update of the most relevant voltage-gated Na^+^\K^+^ ion channels as cellular targets and discuss the possibility of using scorpion venom and toxins for anticancer therapy.

## Ion Channels and Cancer

Ion channels are critical regulators of cellular homeostasis in excitable and non-excitable cells, regulating vital physiological processes, such as electrical signal transmission, gene expression, cell signaling pathways, hormonal secretion, learning, and memory (Bates, 2015). During oncogenic transformation, cancer cells acquire aberrant characteristics with respect to their normal counterparts, which represent the core of cancer hallmarks, such as self-sustained proliferation, tumor progression, angiogenesis, metastasis, and apoptosis resistance (Bates, 2015; Prevarskaya et al., 2018). Many genes encoding ion channels are targets of oncogenic transformation, as previously reported (Prevarskaya et al., 2018). In turn, these gene products contribute to the development of one or more cancer hallmarks, promoting the transition to a more aggressive cancer phenotype; this is exemplified by the positive correlation between ion channel overexpression and functional dysregulation with tumor progression, invasion, and metastasis (Litan and Langhans, 2015; Prevarskaya et al., 2018). The amount of evidence showcasing abnormal ion channel activity linked to carcinogenesis, cancer migration, and invasion has led to consideration of cancer as a channelopathy (Litan and Langhans, 2015; Prevarskaya et al., 2018).

In cancer, the expression changes of ion channels can be related to early diagnosis, prediction of disease aggressiveness, or as markers that allow monitoring of the response to treatment (Lastraioli et al., 2015; Kischel et al., 2019). Different ion channel subfamilies have been associated with a great variety of cancers from different histological origins and even with particular stages of cancer initiation and progression (Rao et al., 2015; Kischel et al., 2019).

In the present article, we focus on voltage-dependent K^+^- and Na^+^-channels as these are the main targets of scorpion venom in prey capture and self-defense behaviors (Quintero-Hernández et al., 2013).

## K^+^-Channels in Cancer

**Figure 1:**
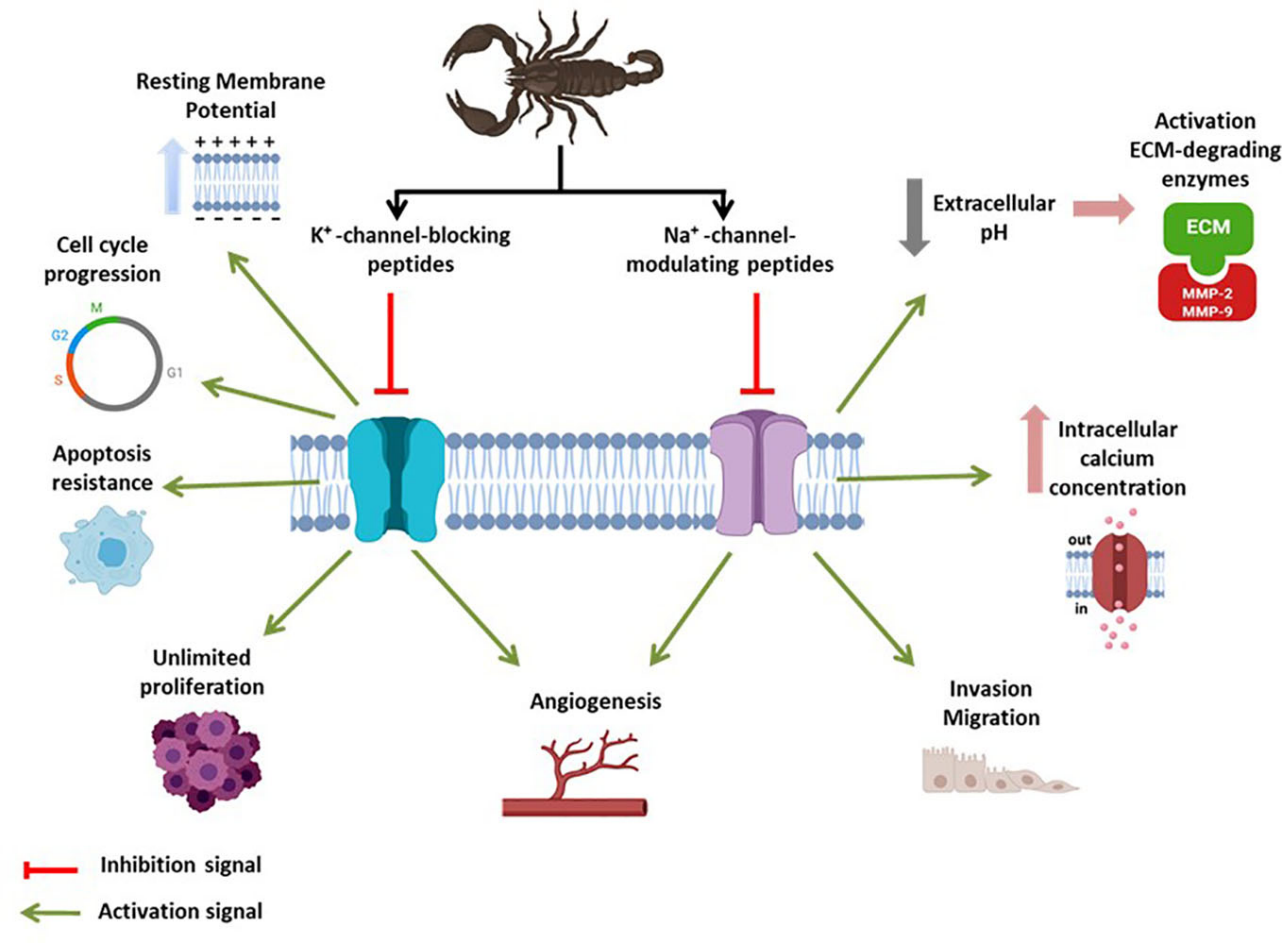
The global effect of scorpion toxins on cancer-related voltage-gated K^+^/Na^+^-channels. An “activation signal” (green) indicates the pathological feature of ion channel activity in the context of cancer development. An “inhibition signal” (red) indicates inhibitory action of scorpion toxins, meaning cancer-hallmark inhibition.

K^+^-channels control K^+^ permeability, and play crucial roles in both excitable and non-excitable cells (Kuang et al., 2015). Voltage-dependent K^+^-channels constitute the largest and most diverse group of voltage-gated ion channels expressed in cells and comprise a pore-forming subunit (K_V_α subunit) that may associate with auxiliary K_V_β subunits (Tian et al., 2014; Kuang et al., 2015). The K_V_β subunits modify ion channel function and/or localization and increase the diversity of physiological roles associated with these ion channels, with implications in health and disease (Tian et al., 2014; Serrano-Novillo et al., 2019). The scientific literature shows a considerable amount of information indicating the role of K^+^-channels in cell proliferation, cancer progression (Wulff and Castle, 2010; Ouadid-Ahidouch et al., 2016), and migration (Chow et al., 2018), and at least four different mechanisms have been proposed (**Figure 1**), and discussed in-depth in recent dedicated reviews (Huang and Jan, 2014; Pardo and Stühmer, 2014).

In cancer cells, there are significant alterations in the expression of K^+^-channels, which is manifested not only by the increase in their total expression, but also in the relative proportion of their different subtypes (Jiang et al., 2017; Zavala et al., 2019). The most prominent ion channel subfamilies present in primary tumors and metastases include Kv, Ether-à-go-go (EAG), and K_Ca_ (Tian et al., 2014; Kuang et al., 2015). Kv10.1, Kv11.1, K_Ca_1.1, and Kv1.3 are the most investigated ion channels, due to their cancer hallmark-related properties. Their implication in preclinical and clinical behavior related to different cancer stages raises them as potential targets for therapy (**Table 1**) (Comes et al., 2015; Prevarskaya et al., 2018).

**Table 1:** Main characteristics of the most studied cancer-related K^+^/Na^+^-channels and their recognized modulating toxins.

Ion channel	Characteristics		Localization		Expression level		Biological Activity*		Scorpion toxin modulators	Ref
			Normal tissue	Cancer tissue	Normal tissue	Cancer tissue	Normal tissue	Cancer tissue		
K_V_11.1	Voltage-dependent activation-inactivation/rapid delayed rectifier		Colon (smooth muscle), pancreas, uterus, kidney, blood, brain, heart	Leukemia, ovarian, lung, breast, colon, gastric, brain, skin, prostate	Low expression	Overexpression	Action potential repolarization (heart), firing frequency and hormone release (endocrine cells), excitability (CNS)	Cell cycle, cell proliferation, apoptosis, migration, invasion	CsEKerg1	(Jehle et al., 2011; Arcangeli and Becchetti, 2015; Cubeddu, 2016; Goversen et al., 2019)
K_V_10.1	Voltage-gated non-inactivating delayed rectifier channel/Calmodulin inhibition		Hypothalamus, hippocampus, cerebral cortex, cerebellum, olfactory nerve	Cervix, lung, breast, colon, ovarian, neuroblastoma, liver, prostate, glioma, gastric, head and neck, squamous cell carcinoma	Low expression	Overexpression	Activation of? excitable cells, signal transduction, hormone secretion regulation, intracellular osmoregulation	Ion flux-independent mechanism for migration, cell cycle G1-G2/M progression, intracellular pathways, cell proliferation, tumor progression	κ-Hefutoxin 1	(Martínez et al., 2015; Ouadid-Ahidouch et al., 2016; Cázares-Ordoñez and Pardo, 2017; Wang et al., 2017)
K_V_1.3	K_V_1.3 (cell membrane)	Voltage-dependent activation-inactivation/delayed rectifier channel	Hypothalamus, olfactory bulb, immune cells, kidney, colon	Breast, colon, smooth muscle, skeletal muscle, lymph node, B cells	Low expression	Overexpression	Resting membrane potential setting, signal transduction, cell proliferation, volume regulation	Proliferation through driving force for Ca^2+^, migration	KAaH1, KAaH2, charybdotoxin, margatoxin, maurotoxin	(Pérez-Verdaguer et al., 2016; Leanza et al., 2017; Venturini et al., 2017; Serrano-Albarrás et al., 2018)
	mK_V_1.3 (inner mitochondrial membrane)	Voltage dependent activation-inactivation				Overexpression/Low expression in apoptotic resistant cancers	Mitochondrial membrane potential regulation	Apoptosis, ROS production, cell proliferation, intracellular signaling pathways		(Leanza et al., 2017; Checchetto et al., 2019)
K_V_1.1	Voltage-dependent activation-inactivation		Central and Peripheral Nervous Systems (hippocampus, cerebellum, neocortex, peripheral nerves)	Glioblastoma, neuroblastoma, breast, colon adenocarcinoma, lung, cervix	Low expression	Overexpression	Control of firing frequency, regulate action potential repolarization, regulate neurotransmitter release	Tumor progression, mitochondrial metabolism, migration	KAaH1, KAaH2	(Leanza et al., 2014; Liu et al., 2019; D'Adamo et al., 2020)
K_Ca_1.1	Voltage-dependent activation/Ca^2+^-modulated		skeletal muscles, nervous system, epithelium endocrine/exocrine glands, endothelial vascular cells, smooth muscle cells	Somatostatinoma, endometrial, prostate, pituitary, breast, glioblastoma, neuroblastoma	Low expression	Overexpression	Modulation of calcium-signaling processes	ERK1/2 signaling, proliferation, migration, metastasis, apoptosis	Iberiotoxin, charybdotoxin	(Oeggerli et al., 2012; Contreras et al., 2013; Du et al., 2016; Li et al., 2018)
nNaV1.5	Voltage-dependent activation–inactivation		Skeletal muscle, heart	Breast	Adult variant (NaV1.5)	Neonatal variant (nNaV1.5)	Action potential generation and propagation	Migration, invasion, metastasis	unknown	(Driffort et al., 2014; Nelson et al., 2015a; Nelson et al., 2015b; Yamaci et al., 2017; Djamgoz et al., 2019)
NaV1.6	Voltage-dependent activation–inactivation		CNS neurons	Cervix, colorectal, astrocytoma	Low expression	Overexpression	Action potential generation and propagation	Migration, invasion, metastasis	Cn2, AaHIV	(Lopez-Charcas et al., 2018; Guan et al., 2018)
NaV1.7	Voltage-dependent activation–inactivation		PNS neurons, adrenal gland, endocrine pancreatic cells	Prostate, lung, gastrointestinal tract	Low expression	Overexpression	Action potential generation and propagation	Migration, invasion, metastasis	unknown	(Campbell et al., 2013; Shan et al., 2014; Xia et al., 2016; Chen et al., 2019)

*Referred to both positive and negative correlation respect to ion channel-regulated physiological and biological activity.

K_V_11.1 (also known as the human Ether-à-go-go (hERG) channel) is probably the most studied ion channel in the EAG subfamily. In normal healthy tissues, its expression is usually low. In contrast, this ion channel is expressed in a higher proportion in leukemia, ovarian, lung, and breast cancer cells, among others (Jehle et al., 2011). K_V_11.1 channels have notable participation in the cell cycle and appear as regulators of apoptosis and cell proliferation in cancer cells (Staudacher et al., 2014; Arcangeli and Becchetti, 2015). In the heart, Kv11.1 is key for cardiac repolarization and therefore, its off-target inhibition induces long QT syndrome. Thus, safety pharmacological studies include K_V_11.1 channel assays as the primary test, decreasing its practical impact as an anticancer therapy-related target (Goversen et al., 2019).

K_V_10.1 channel is selectively expressed in brain areas (**Table 1**). However, this channel is overexpressed in more than 70% of tumors and in cancer cell lines from the cervix, lung, breast, ovary, neuroblast, liver, prostate, glial cells, and gastrointestinal tract (Martínez et al., 2015; Wang et al., 2017). Moreover, its crucial role in tumorigenesis, cell signaling, cell cycle, and tumor growth has been recognized (Ouadid-Ahidouch et al., 2016). Different experimental approaches have demonstrated the relationship between K_V_10.1 channel blockage and anticancer effects, including induction of apoptosis, inhibition of cell proliferation, and delay in tumor growth (Cázares-Ordoñez and Pardo, 2017), suggesting that this channel is a promising candidate as a tumor and therapeutic marker in oncology.

K_Ca_1.1 channel is ubiquitously expressed in human tissues such as skeletal muscle and the nervous system, with the exception of cardiac myocytes. K_Ca_1.1 channels regulate calcium influx into cells and thereby modulate Ca^2+^-signaling processes (Contreras et al., 2013). This channel is overexpressed in cancer cell lines from prostate, glia, breast, pancreas, and endometrium (**Table 1**) (Du et al., 2014; Du et al., 2016; Klumpp et al., 2016; Li et al., 2018; Noda et al., 2020). In the prostate, K_Ca_1.1 channel overexpression regulates proliferation and migration (Du et al., 2016) and in breast cancer, its overexpression has been associated with advanced tumor stage, high tumor cell proliferation, and poor prognosis (Oeggerli et al., 2012).

K_V_1.3 channel is mostly expressed in neurons and immune cells (Pérez-Verdaguer et al., 2016). It is located at the plasma membrane, sets the resting membrane potential (RMP) and regulates cell proliferation and cell volume. Furthermore, this channel is also located in the inner mitochondrial membrane (mK_V_1.3), where it plays a role in apoptotic signaling (Teisseyre et al., 2019) (**Table 1**). Overexpression of K_V_1.3 channels is observed in breast, colon, smooth muscle, skeletal muscle, and lymph node cancers (Teisseyre et al., 2015; Teisseyre et al., 2019). Its plasma membrane expression is associated with controlling cell proliferation by inducing a transitory hyperpolarization necessary to augment the driving force for Ca^2+^ influx during G1/S progression (Serrano-Albarrás et al., 2018). Moreover, mK_V_1.3 channels play a role in drug-induced apoptosis by mechanisms that sensitize cancer cells (Pérez-Verdaguer et al., 2016). The potential role of K_V_1.3 channels as cancer therapy targets has been recently evidenced in *in vitro* and *in vivo* experimental models of glioblastoma, melanoma, and pancreatic adenocarcinoma, where mK_V_1.3 inhibition induces apoptotic cell death *in vitro* (Leanza et al., 2017; Venturini et al., 2017; Checchetto et al., 2019). All these pieces of evidence promoted K_V_1.3 channels as attractive potential molecular targets in both cancer diagnostics and therapy (Comes et al., 2015; Prevarskaya et al., 2018).

Notwithstanding that the ion channels mentioned above represent some of the most prominent ones in cancer; other voltage-gated ion channels linked to cancer proliferation and progression are upregulated in some tumors and have been described in dedicated reviews (Huang and Jan, 2014; Serrano-Novillo et al., 2019).

## Na^+^-Channels in Cancer

Voltage-dependent sodium channels (VGSC) are transmembrane proteins that are generally expressed in excitable cells, although they are also found, to a limited extent, in non-excitable cells (Catterall, 2012; Erickson et al., 2018). There are nine pore-forming α-subunits of sodium channels, Na_V_1.1-Na_V_1.9, encoded by the genes SCN1A-SCN11A. The pore-forming α-subunit comprises four highly similar transmembrane domains (I-IV), each composed of six transmembrane segments (S1–S6). The first four transmembrane segments of each domain constitute the voltage sensor domain, and the last two form the pore domain (Catterall, 2012). The α-subunit properties can be modulated in a subtype-specific manner, by association with one or more than one smaller auxiliary β-subunit (Na_V_β_1–4_); conferring tissue-specific expression patterns, varying voltage dependent activation and inactivation, and increasing functional channel density at the plasma membrane (Catterall, 2017).

The oncogenic transformation of VGSC can contribute to the development of one or more cancer hallmarks, promoting the transition to more aggressive cancer phenotypes, as previously reported (Prevarskaya et al., 2018); this is particularly exemplified by the positive correlation between VGSC overexpression and functional dysregulation with invasion/migration and metastatic potential (Andrikopoulos et al., 2011; Djamgoz et al., 2019; Mao et al., 2019) (**Table 1**).

Proliferating and cancer cells show a RMP between -10 to -50 mV, compared to normal and non-proliferating cells (-50 to -90 mV) (Yang and Brackenbury, 2013). This RMP range fits with the window current range for VGSC, meaning that although the majority of VGSCs will be inactivated, the small percentage of non-inactivated channels will lead to a persistent Na^+^-current, increasing the [Na]_i_ (Yang and Brackenbury, 2013). The augmented intracellular Na^+^ concentration leads to an increased intracellular Ca^2+^ concentration, either by promoting the reverse mode of the Na^+^/Ca^2+^ exchanger (NCX) or by inducing plasma membrane depolarization and consequent activation of voltage-sensitive Ca^2+^ channels (VGCC) (Patel and Brackenbury, 2015; Roger et al., 2015). Both mechanisms, driven directly or indirectly by VGSC, might be considered relevant for cancer migration and invasion. However, there are very few reports providing experimental evidence about the functional link between VGSC, NCX, and VGCC (Besson et al., 2015; Angus and Ruben, 2019; Rodrigues et al., 2019) and this aspect needs broader investigation.

A hallmark of a tumor´s extracellular space is a more acidic environment than in normal healthy tissues (pH 6.2–6.8 instead of pH 7.2–7.4), as a consequence of the predominant glycolytic metabolism of cancer cells; this particular extracellular environment enhances the degradation of the extracellular matrix by favoring Cathepsin B and S activation, and thus, promotes cell migration (Besson et al., 2015; Angus and Ruben, 2019). This extracellular acidification is dependent on Na^+^/H^+^ exchanger 1 (NHE1), which in turn depends on the [Na^+^] transmembrane gradient (Besson et al., 2015; Angus and Ruben, 2019). Given the increased [Na]_i_, a reduced NHE1 activity should be expected; however, two hypotheses have been suggested to explain this apparent contradiction. i) that these channels allosterically regulate NHE1 by inducing a higher rate of H^+^ extrusion at neutral pHi ranges, and ii) that the expression of VGSC in late endosome vesicles is responsible for the extra-acidification of these vesicles (Besson et al., 2015; Angus and Ruben, 2019). In this last scenario, the extracellular acidic environment would be a consequence of vesicle release.

Tetrodotoxin (TTX) is a toxin, mainly associated with fishes of the *Tetraodontidae* family, that specifically blocks a subgroup of VGSCs and inhibits the migration and invasion of cancer cells, indicating that cell motility requires Na^+^-channel activity (Nelson et al., 2015a) a feature mainly associated with overexpression of the neonatal variants of Na_V_1.5 (nNa_V_1.5), Na_V_1.6, and Na_V_1.7 (Roger et al., 2015; Mao et al., 2019).

nNa_V_1.5 overexpression was initially identified in the metastatic human breast cancer cell line MDA-MB-231 and breast biopsy samples (**Table 1**) (Yamaci et al., 2017). Later, the same positive correlation was found between the expression of nNa_V_1.5 channels and the high invasive potential of cancer cells from diverse histological origins (Djamgoz et al., 2019), suggesting that the overexpression of nNa_V_1.5 channel is necessary and sufficient to increase the metastatic potential of cancer cells (Nelson et al., 2015b).

Na_V_1.6 is overexpressed in cervical cancer biopsies, cancer cell lines, and primary cultures positive for the human papillomavirus (**Table 1**). In these cases, a Na_V_1.6 splice variant with preferential cytoplasmatic localization is expressed (Lopez-Charcas et al., 2018). Overexpression of Na_V_1.6 protein is associated with invasive status in cervical cancer and low-grade astrocytoma, mediated through increased MMP-2 activity (Lopez-Charcas et al., 2018; Guan et al., 2018).

Na_V_1.7 is ectopically expressed in particular types of cancers (**Table 1**) (Campbell et al., 2013; Xia et al., 2016; Chen et al., 2019). In gastric cancer, this channel is associated with poor patient outcomes by promoting cell invasion through the modulation of H^+^ efflux (Xia et al., 2016). In rat prostate cancer, Na_V_1.7 channel activity promotes the activation of p38/NF-κβ, and Rho GTPase signaling pathways as a linking node for controlling cellular motility, cell adhesion, and vesicular trafficking (Chen et al., 2019). In non-small cell lung cancer, the Na_V_1.7 channel is overexpressed in metastatic cells by more than 60% when compared to their non-metastatic counterparts (Campbell et al., 2013).

Independent of their function as auxiliary subunits, Na_V_β_1-3_ are overexpressed in different cancers and have been associated with increased cellular motility, invasion, and metastasis (O'Malley and Isom, 2015; Bouza and Isom, 2018). Additionally, Na_V_β_1_ has been linked to tumor growth, increase of vascular endothelial growth factor secretion, and angiogenesis (O'Malley and Isom, 2015; Bouza and Isom, 2018). In contrast, Na_V_β_3_ functions as a tumor suppressor by inducing p53-dependent apoptosis when overexpressed (Bouza and Isom, 2018). Thus, the Na_V_β-subunits are interesting and poorly explored potential targets for cancer therapy, needing an in-depth investigation to identify their complete clinical and physiopathological relevance.

Overall, VGSCs and Na_V_β are up-regulated in numerous types of metastatic cancer cells and play important roles in regulating cell migration and invasion in solid tumors. Therefore, they can be considered as key regulators of cancer development and the metastatic cascade (Mao et al., 2019). The noncanonical activity of VGSC that regulates other cancer hallmarks (*i.e.*, cell proliferation) is scarcely understood and needs to be investigated with more detail (Black and Waxman, 2013).

## Scorpion Venom and Their Toxins in Cancer

Worldwide, there are more than 2,200 scorpion species, grouped in 19 families (Ward et al., 2018). The scorpion venom is a complex mixture containing a great variety of proteins with molecular weights between 3 kDa and 90 kDa, which constitute most of the components. The main biological activity of the scorpion venom is due to the presence of low molecular weight peptide toxins of basic nature, which are highly cross-linked (3–4 disulfide bridges) (Quintero-Hernández et al., 2013; Kuzmenkov et al., 2015). These peptides exhibit different pharmacological and toxicological activities (Quintero-Hernández et al., 2013; Kuzmenkov et al., 2015). Until now, only a few scorpion species have been experimentally tested as anticancer agents, mainly for cancer cells from solid tumors and to a lesser extent, for hematopoietic cancers (Raposo, 2017).

In only two cases (*B. martensii* and *R. junceus*), the scientific results correlate with the experiences in traditional medicine and with the low toxicity recognized in toxicological experiments in mice (Wang and Ji, 2005; Diaz-Garcia et al., 2019a; Díaz-García et al., 2019b). The anticancer effect of *B. martensii* scorpion venom has been tested successfully against human glioma U251-MG by using rodent xenograft models (Wang and Ji, 2005). Likewise, *in vivo* toxicological studies have been carried out, using *R. junceus* venom administered through intraperitoneal (10 mg/kg) or oral (2,000 mg/kg) routes, and toxic effects have not been observed (Garcia-Gomez et al., 2011; Lagarto et al., 2020). Pharmacokinetic and biodistribution studies carried out on breast tumor-bearing mice administered with a single dose (12.5 mg/kg), by intravenous or oral routes, showed that medium residence time (MRT) of venom in tumor tissue was higher than in the remaining organs tested, suggesting a high selectivity for tumor tissue, adding to their antitumor effect (Diaz-Garcia et al., 2019a). Additionally, breast tumor-bearing mice injected intraperitoneally with ten consecutive doses of *R. junceus* venom (3.2 mg/kg), showed reduced tumor progression and reduction of Ki67 and CD31 tumor markers, confirming its anticancer potential (Díaz-García et al., 2019b). Two additional scorpion species, *Androctonus amoreuxi* (Salem et al., 2016) and *Leiurus quinquestriatus* (Al Asmari and Khan, 2016), have been tested with some favorable *in vivo* anticancer effects, even though both are two of the most dangerous species (Ward et al., 2018). These overall promising results have focused the scientific research on the isolation and identification of the components responsible for the anticancer effects of scorpion venoms.

Peptides recognizing K^+^- and Na^+^-channels are prominent in scorpion venoms, constituting more than 75% of all peptide/proteins (de Oliveira et al., 2018; Cid-Uribe et al., 2019). Most peptides recognizing K^+^ channels are pore-blocking peptides and some of them have been studied in the context of cancer (**Table 1**). For example, KAaH1, a K_V_1.1 and K_V_1.3 blocker, and KAaH2, a K_V_1.1 blocker, both derived from the *Androctonus australis Hector* venom, have shown anticancer potential (Aissaoui et al., 2018). KAaH1 inhibits migration and adhesion of different cancer cells, whereas KAaH2 inhibits the proliferation of gliomas (Aissaoui et al., 2018). Evidence indicates that iberiotoxin inhibits cell proliferation, migration, and invasion in breast and endometrial cancer cell lines, due to its blocking effects on BK channels (Schickling et al., 2015; Li et al., 2018); while charybdotoxin, a known blocker of K_Ca_3.1, K_V_1.3, and BK channels, inhibits proliferation and cell cycle progression in pancreatic and endometrial cancer cell lines (Jager et al., 2004; Schickling et al., 2015; Li et al., 2018). Both toxins were isolated from the *Leiurus quinquestriatus* scorpion. Similarly, margatoxin (MgTX), a peptide isolated from *Centruroides margaritatus*, is a selective K_V_1.3-blocker that reduces cell proliferation, and tumor progression, decreases the expression of cell cycle regulators and increases the expression level of proapoptotic proteins in cancer experimental models (Jang et al., 2011). CsEKerg1 toxin, from the *Centruroides sculpturatus* scorpion has been evaluated as a hERG current inhibitor in an *in vitro* cancer model, suggesting its potential use in Kv11.1 channel-overexpressing cancer cells (Nastainczyk et al., 2002); this result opens a window of opportunity for other Kv11.1-blocking toxins described until now (Jimenez-Vargas et al., 2012). κ-Hefutoxin 1 from *Heterometrus fulvipes* scorpion venom (Moreels et al., 2017) has been identified as the first toxin recognizing K_V_10.1 channels, without affecting other voltage-gated K^+^-channels (Moreels et al., 2017). Moreover, maurotoxin isolated from *Scorpio maurus palmatus* scorpion can block various potassium channels, including SK, IK, K_V_1.1, and K_V_1.3, some of which have been recognized as cancer-related ion channels (Castle et al., 2003). Tapamin, a toxin isolated from the *Mesobuthus tamulus* scorpion, can block some cancer-related ion channels, such as SK and K_Ca_3.1, and exerts a cytotoxic effect on cancer cells (Pedarzani et al., 2002; Ramirez-Cordero et al., 2014).

Although Na^+^-channel-modulating peptides represent the highest percentage among all scorpion venom-derived toxins (Cid-Uribe et al., 2019), the identification of scorpion venom peptides that interact with metastatic-related Na^+^ channels has been difficult, and only three cases have been identified (**Table 1**). Cn2, a β-toxin from *Centruroides noxius Hoffmann* scorpion venom, modulates Na_V_1.6 activity in F11 neuroblastoma cells (Escalona et al., 2014). In cell culture, Cn2 reduces proliferation by increasing cells at the SubG1 and G0/G1 stages, leading to apoptosis induction (Escalona et al., 2014). This toxin binds to the receptor site 4, located in the S3–S4 and S1–S2 extracellular loops of the VGSC channel domain II, enhancing channel activation by shifting the voltage-dependence of channel activation to the left, as a consequence of voltage-sensor trapping (Cestele et al., 1998), and reducing the Na^+^ current peak amplitude (Pedraza Escalona and Possani, 2013). AGAP, isolated from *Buthus martensii*, is an α-toxin that interacts with Na^+^-channels. Evidence suggests that AGAP affects the translation of the Na_V_β1 subunit in cancer cells and has been successfully evaluated against Ehrlich ascites tumor and S-180 fibrosarcoma models *in vivo*. Furthermore, this peptide can inhibit cancer cell stemness, epithelial-mesenchymal transition (EMT), migration, and invasion in MCF-7 and MDA-MB-231 human breast cancer cells *in vitro* and tumor growth *in vivo* (Guo et al., 2016; Kampo et al., 2019). Finally, AaHIV toxin, isolated from *Androctonus australis* venom, is a Na^+^ channel-modulating toxin active against cancer cells (BenAissa et al., 2019). AaHIV can interact with the extracellular loops of segments S1–S2 in the voltage sensor domain, prolonging the inactivation recovery time of Nav1.6 channels, and inhibiting cancer cell proliferation in a dose-dependent manner (BenAissa et al., 2019). Unlike anti-migratory and anti-metastatic properties, the antiproliferative properties of Na^+^-channel-interacting scorpion toxins represent an unexpected feature that should be deeply investigated. There is no doubt that scorpion venom peptide toxins inhibit the functional activity of voltage-gated K^+^/Na^+^-channels, reducing their impact on the hallmark of cancer (**Figure 1**).

It is worth mentioning that Chlorotoxin is the only toxin from scorpion venom that has been successfully evaluated in cancer preclinical and clinical trials (Cohen-Inbar and Zaaroor, 2016; Mahadevappa et al., 2017; Cohen et al., 2018). However, this toxin recognizes voltage-dependent Cl^-^ channels (Dardevet et al., 2015), which was not within the scope of this review.

## Concluding Remarks

Evidence indicates that upregulation of voltage-dependent K^+^ and Na^+^ channels is linked to cancer hallmarks. Thus, they have become key player as new alternatives to be used as diagnostic, prognostic, and therapeutic targets in cancer. Scorpion venoms contain small peptides acting either at the cell membrane or intracellularly, and even cross the blood-brain barrier. The mechanisms of action of scorpion venom toxins described here, related to ion channel-modulating effects, give new insights to the plethora of potential new mechanisms of action that could be discovered from scorpion venom peptides. Laboratories dedicated to scorpion venom research have usually described the anticancer effects of scorpion venom and/or components for the first time; far away from the anticancer drug development programs and their resources. There is no doubt that the inclusion of these natural products, such as plant extracts, as part of the anticancer drug discovery programs, might increase the arsenal of active components as potential new drugs against relatively new targets. Importantly, the interaction of both research areas might represent a substantial qualitative leap that could open a highway of promising alternatives to be used as adjuvant therapeutic approaches or conventional treatment in anticancer therapy.

## Author Contributions

Both authors contributed equally to the writing and preparation of the manuscript.

## Funding

The Millennium Nucleus of Ion Channel-Associated Diseases (MiNICAD) is a Millennium Nucleus supported by the Iniciativa Científica Milenio of the Ministry of Economy, Development, and Tourism (Chile). This work was supported by Vicerrectoría de Investigación y Desarrollo, Universidad de Chile (VID‐Enlace, ENL24/19).

## Conflict of Interest

AD-G works for LifEscozul Chile SpA.

The remaining author declares that the research was conducted in the absence of any commercial or financial relationships that could be construed as a potential conflict of interest.
